# Thykamine™: A New Player in the Field of Anti-Inflammatory Drugs

**DOI:** 10.3390/biomedicines13122938

**Published:** 2025-11-29

**Authors:** Charles Lynde, Louis Flamand, Vincent McCarty, John Sampalis

**Affiliations:** 1 Department of Medicine, University of Toronto, Toronto, ON M5S 3H2, Canada; 2 Lynderm Research Inc., Probity Medical Research, Markham, ON L3P 1X3, Canada; 3 Department of Microbiology, Infectious Disease and Immunology, Faculty of Medicine, Université Laval, Quebec City, QC G1V 0A6, Canada; 4 Department of Surgery, McGill University, Montreal, QC H3G 1A4, Canada

**Keywords:** cytokine, chemokine, inflammation, topical, anti-inflammatory, botanical

## Abstract

**Background/Objectives**: Persistent inflammation driven by cytokines/chemokines plays a crucial role in the pathogenesis of numerous chronic inflammatory and autoimmune conditions, including rheumatoid arthritis, atopic dermatitis, and ulcerative colitis. Current therapeutic agents often present limitations due to adverse effects. Thykamine™, a new plant-derived multi-target drug, has demonstrated promising anti-inflammatory effects and a favorable safety profile in clinical settings. This study aimed to compare the in vitro chemokine-inhibitory potency of Thykamine™, a novel plant-derived anti-inflammatory compound, with that of six marketed corticosteroid and non-steroidal agents. **Methods**: This study compared the in vitro potency of Thykamine™ against widely prescribed anti-inflammatory agents, including corticosteroids (betamethasone, clobetasol, hydrocortisone, prednisone) and non-steroidal therapies (crisaborole, pimecrolimus). Potency was assessed by measuring the inhibition of key pro-inflammatory chemokines: MCP-1, MIP-1α, MIP-1β, and RANTES in lipopolysaccharide-stimulated U937 cells. **Results**: Area-under-the-curve (AUC) analyses confirmed that Thykamine™ inhibited secretion of the chemokines MCP-1, MIP-1α, and MIP-1β with significantly greater potency than all other agents tested. Thykamine™ also suppressed secretion of RANTES similarly to prednisone and significantly more than betamethasone, clobetasol, hydrocortisone, and pimecrolimus but less than crisaborole due to crisaborole’s elevated potency when administered at high concentration. **Conclusions**: Overall, Thykamine™ showed significantly greater or comparable inhibitory potency, particularly at lower concentrations, without evidence of cytotoxicity. These findings underscore the potential of Thykamine™ as a potent, multi-target anti-inflammatory therapy, which could offer substantial clinical advantages by effectively controlling chemokine-mediated inflammation with potentially fewer adverse effects. The results of this study support the need for evaluation of the clinical therapeutic efficacy of Thykamine™ in a wide range of autoimmune conditions.

## 1. Introduction

Inflammation is a fundamental protective mechanism of the immune system. Its primary functions are the recruitment of effector cells for elimination of pathogens and promotion of tissue repair. Inflammation begins when innate immune cells detect infection or tissue damage through pattern recognition receptors (PRRs), such as Toll-like receptor-4 (TLR4). These PRRs are found on the surfaces of phagocytic cells, including macrophages, neutrophils, and dendritic cells [1]. PRRs recognize pathogen- or damage-associated molecular patterns (PAMPs/DAMPs) and activate transcription factors, including nuclear factor kappa-light-chain-enhancer of activated B cells (NF-κB) [2]. NF-κB and other transcription factors then drive transcription of an array of target genes to induce the production and secretion of pro-inflammatory cytokines. These cytokines coordinate the activation and recruitment of immune cells to the site of infection or tissue damage.

Chemokines are cytokines that attract immune cells to an affected area. Chemokines upregulated by NF-κB include monocyte chemoattractant protein-1 (MCP-1), also known as C-C chemokine ligand (CCL) 2, macrophage inflammatory protein-1 alpha (MIP-1α, or CCL3), macrophage inflammatory protein-1 beta (MIP-1β, or CCL4), and regulated on activation, normal T cell expressed and secreted (RANTES, or CCL5). These chemokines promote migration of immune cells via binding C-C chemokine receptors (CCRs). MCP-1 preferentially binds CCR2 [3]; MIP-1α mainly binds CCR1 and CCR5 [4]; and both MIP-1β and RANTES primarily bind CCR5 (Table 1) [5,6]. In addition to driving chemotaxis, chemokines can promote other inflammatory activity [7]. MCP-1, for example, also stimulates the production of pro-inflammatory cytokines, such as interleukin (IL)-1 [7], IL-6 [7,8], and tumor necrosis factor (TNF)-α [9], and enhances the maturation of monocytes into M2 macrophages [8].

Though vital to a healthy immune response, persistent production of chemokines can lead to chronic immune response. This can damage healthy tissues and disrupt normal tissue function, causing autoimmune disease. In various autoimmune conditions, the levels of MCP-1, MIP-1α, MIP-1β, and RANTES are higher in patients versus healthy controls (Table 2). Chemokine levels have also been found to correlate with disease severity [10,11].

**Table 1:** Characterization of select chemokines.

Characteristic	MCP-1	MIP-1α	MIP-1β	RANTES
**Main receptors**				
CCR1		✓ [4]		✓ [6]
CCR2	✓ [3]			
CCR3				✓ [6]
CCR5		✓ [4]	✓ [5]	✓ [6]
**Associated immune cell types**				
Monocytes	✓ [12]	✓ [13]	✓ [14]	✓ [15]
Neutrophils	✓ [16]	✓ [16]	✓ [14]	✓ [17]
CD4+, CD8+, and NK T lymphocytes	✓ [18,19]	✓ [18,20]	✓ [18,19,21]	✓ [18,19]
Dendritic cells	✓ [22]	✓ [23]	✓ [23]	✓ [24]
Eosinophils	✓ [25]	✓ [26]	✓ [27]	✓ [26]

✓ Indicates there is published evidence of receptor binding or interaction with an immune cell type for a given chemokine. Numbers in square brackets correspond to published evidence. MCP-1: Monocyte Chemoattractant Protein-1; MIP-1α: Macrophage Inflammatory Protein-1 alpha; MIP-1β: Macrophage Inflammatory Protein-1 beta; RANTES: Regulated on Activation, Normal T Cell Expressed and Secreted; CCR: C-C Chemokine Receptor; CD: Cluster of Differentiation; NK: Natural Killer.

**Table 2:** Elevated levels of MCP-1, MIP-1α, MIP-1β, and RANTES in patients versus healthy controls for select autoimmune diseases.

Disease	Sample Type	Reference	MCP-1	MIP-1α	MIP-1β	RANTES
Rheumatoid arthritis	Serum	[28]	✓	✓	✓	✓
Psoriatic arthritis	Serum	[10,29]	✓	✓	✓	✓
Osteoarthritis	Synovial fluid	[13,30,31]	✓	✓	✓	✓
Atopic dermatitis	Serum	[32]	✓		✓	✓
Asthma	Bronchoalveolar lavage fluid	[33]	✓	✓		✓
Inflammatory bowel disease (IBD; ulcerative colitis, Crohn’s disease)	Intestinal mucosa	[34,35]	✓	✓	✓	✓
Osteoporosis	Serum	[36]	✓	✓	✓	✓
Metabolic-dysfunction-associated steatohepatitis (MASH)	Plasma/serum	[37,38]	✓	✓	✓	✓
Systemic sclerosis	Serum	[39,40]	✓	✓	✓	✓
Systemic lupus erythematosus	Serum	[41]			✓	✓
Severe COVID-19	Serum	[42]	✓	✓	✓	✓

✓ Indicates there is published evidence of elevated levels of a given chemokine. Numbers in square brackets correspond to published evidence. MCP-1: Monocyte Chemoattractant Protein-1; MIP-1α: Macrophage Inflammatory Protein-1 alpha; MIP-1β: Macrophage Inflammatory Protein-1 beta; RANTES: Regulated on Activation, Normal T Cell Expressed and Secreted.

Management of autoimmune diseases relies on anti-inflammatory or immunomodulatory agents. These include corticosteroids, such as clobetasol, betamethasone, hydrocortisone, and prednisone, and/or non-steroidal therapies, such as crisaborole or pimecrolimus (Table 3). The use of these agents is often limited by cost and adverse effects. Prednisone can lead to osteoporosis [43] and weight gain [44]. Oral corticosteroids have been associated with sepsis, venous thromboembolism, and fractures [45]. While postnatal corticosteroid therapy is useful for improving pulmonary function in preterm infants, it can also affect growth, as measured by body weight, height, and head circumference [46]. Fear of adverse events with corticosteroid use is associated with poor treatment adherence [47]. Given these challenges, there is an unmet need for effective anti-inflammatory agents that have fewer side effects.

Thykamine™ is a complex, multi-component botanical extract derived from spinach thylakoid membranes. It comprises fragments of lipid bilayers, which are rich in galactolipids, such as monogalactosyl diacylglycerol (MGDG), digalactosyl diacylglycerol (DGDG), and sulfoquinovosyl diacylglycerol (SQDG); embedded pigments (chlorophylls and carotenoids); and residual proteins and enzymes inherent to thylakoids, which confer superoxide-dismutase-like activity. The extract maintains the partial architecture of membrane segments rather than being fully disassembled molecules; thus, its structure is best thought of as membranous particles loaded with lipids, pigments, and functional enzymatic moieties [58]. Previous in vitro research has found that Thykamine potently inhibits the inflammatory response [58] and offers antioxidant properties, and it can protect cells against reactive oxygen species as well as UV exposure [59,60].

The potential of Thykamine™ as an anti-inflammatory and immune modulating therapy was highlighted in a Phase IIa clinical trial in patients with active mild-to-moderate distal ulcerative colitis (UC) [61] and in a Phase II clinical study in adult patients with mild-to-moderate atopic dermatitis [62]. In atopic dermatitis, 30.8% of patients receiving Thykamine™ achieved the primary endpoint, an IGA score of clear/almost clear and a ≥2-point reduction at day 29, versus 6.7% in the vehicle group (*p* = 0.014). Secondary endpoint results included significant reductions versus vehicle in the body surface area (BSA) affected, pruritus, and patient-oriented eczema measure (POEM). In UC, Thykamine™ rectal enema administered once daily for 2 weeks led to significant reductions in rectal bleeding, fecal lactoferrin, serum TGF-β, and M30 apoptosome biomarkers, with favorable safety with no treatment-related serious adverse events.

The current study compares Thykamine™ to betamethasone, clobetasol, crisaborole, hydrocortisone, prednisone, and pimecrolimus with respect to the in vitro inhibition of MCP-1, MIP-1α, MIP-1β, and RANTES in response to stimulation with lipopolysaccharide (LPS), a pathogen-associated molecular pattern (PAMP) that, when recognized by TLR4, induces inflammatory response [1].

## 2. Materials and Methods

### 2.1. Cell Culture and Experimental Design

To assess the anti-inflammatory effects of Thykamine™ versus existing anti-inflammatory agents, U937 cells (American Type Culture Collection; Manassas, VA, USA) were seeded at a density of 2.95 × 10^5^ cells per well in 24-well tissue culture plates in 900 µL of culture medium [RPMI-1640 (Corning; Manassas, VA, USA), 10% fetal bovine serum (FBS) (Corning; Manassas, VA, USA), and Plasmocin^®^ antibiotics (Invivogen; San Diego, CA, USA)]. Comparator agents were received as powders and dissolved in dimethyl sulfoxide (DMSO) (ThermoFisher Scientific; Burlington, ON, Canada). The dissolved comparator agents and Thykamine™ (water-soluble) were serially diluted in the culture medium to achieve test concentrations. The concentrations of Thykamine™ tested were based on a similar study of another botanical extract’s impact on pro-inflammatory responses to LPS stimulation in U937 cells [63]. Comparator agent test concentrations were based on published literature [64,65,66,67,68] and are presented in Table 4. After four hours of incubation at 37 °C in a 5% CO_2_ atmosphere, diluted agent formulations were added (50 µL per well). DMSO at 0.05% final concentration was included as a control. Following agent addition, cells (except unstimulated controls) were stimulated with 50 µL of LPS (250 ng/mL final concentration) in saline solution (Sigma-Aldrich; St. Louis, MO, USA; L5418). All conditions were run in hexaplicate (*n* = 6).

### 2.2. Cytokine Quantification

After overnight incubation at 37 °C in a 5% CO_2_ atmosphere, cell-free supernatants were collected and immediately assayed using the Pro-CartaPlex™ (Life Technologies; Carlsbad, CA, USA) for the detection of MCP-1, MIP-1α, MIP-1β, and RANTES.

### 2.3. Statistical Analysis

The differences in cytokine inhibition between Thykamine™ and the comparator agents were tested with one-way analysis of variance (ANOVA) and Bonferroni adjustment. The percent inhibition of cytokine production was calculated as the reduction in cytokine levels relative to LPS-stimulated control conditions (mean across 6 wells). The area under the curve (AUC) for inhibition was calculated using the standard trapezoidal rule for each well, and mean AUC values were compared across agents. IBM SPSS Statistics (Version 29.0) was used for all data analyses.

## 3. Results

Stimulation of U937 cells with LPS prompted the release of chemokines into the supernatant, with LPS-stimulated cells secreting more than five times as much MCP-1 and nearly twice as much RANTES versus unstimulated cells. Similarly, cells stimulated with LPS secreted MIP-1α and MIP-1β, both of which were undetectable in the supernatant collected from unstimulated cells. All tested anti-inflammatory agents inhibited the release of all cytokines, except pimecrolimus.

### 3.1. MCP-1

**Figure 1:**
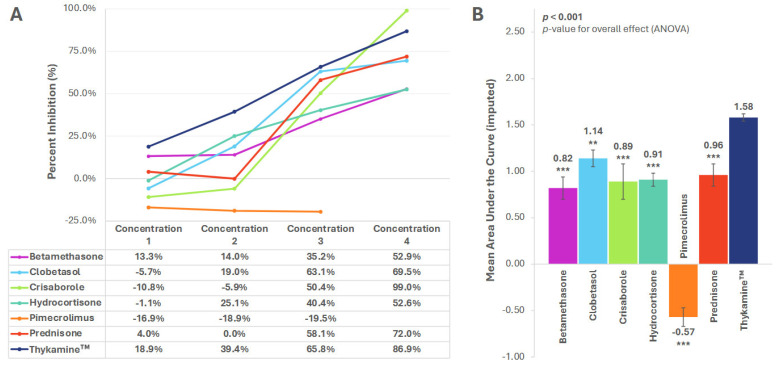
Inhibition of MCP-1 by anti-inflammatory agents. (**A**) Percent inhibition of MCP-1 secretion by anti-inflammatory agents across concentrations; (**B**) Mean area under the curve (AUC) for percent inhibition. AUC imputed for pimecrolimus. *** *p* < 0.001, ** *p* < 0.01. *p*-value for planned paired contrast (ANOVA) versus Thykamine™. All significant differences remained significant after Bonferroni adjustments. Error bars represent standard error of the mean. Six technical replicates per concentration per agent.

Inhibition of MCP-1 was observed with Thykamine™ and all other agents, except pimecrolimus. With each drug, there was a direct association between percent inhibition and concentration level (Figure 1A). Thykamine™ showed higher inhibition of MCP-1 than all other drugs at all concentrations, except for the highest concentration of crisaborole (Figure 1A).

The mean (SE) AUC for Thykamine™ percent inhibition of MCP-1 was 1.58 (0.04) and significantly higher versus betamethasone [0.82 (0.120, *p* < 0.001], clobetasol [AUC 1.14 (0.09), *p* = 0.008], crisaborole [0.89 (0.19), *p* < 0.001], hydrocortisone [0.91 (0.07), *p* < 0.001], pimecrolimus [−0.57 (0.10), *p* < 0.001], and prednisone [0.96 (0.12), *p* < 0.001] (Figure 1B).

### 3.2. MIP-1α and MIP-1β

**Figure 2:**
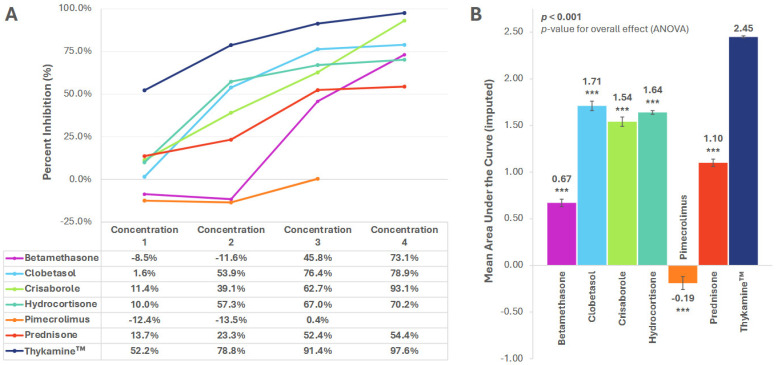
Inhibition of MIP-1α by anti-inflammatory agents. (**A**) Percent inhibition of MIP-1α secretion by anti-inflammatory agents across concentrations; (**B**) Mean area under the curve (AUC) for percent inhibition. AUC imputed for pimecrolimus. *** *p* < 0.001. *p*-value for planned paired contrast (ANOVA) versus Thykamine™. All significant differences remained significant after Bonferroni adjustments. Error bars represent standard error of the mean. Six technical replicates per concentration per agent.

**Figure 3:**
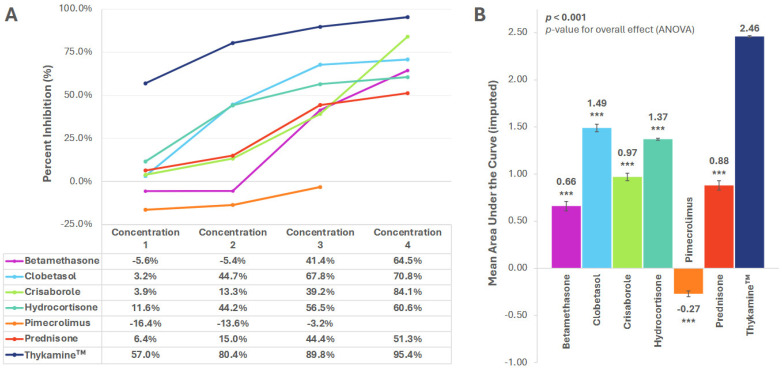
Inhibition of MIP-1β by anti-inflammatory agents. (**A**) Percent inhibition of MIP-1β secretion by anti-inflammatory agents across concentrations; (**B**) Mean area under the curve (AUC) for percent inhibition. AUC imputed for pimecrolimus. *** *p* < 0.001. *p*-value for planned paired contrast (ANOVA) versus Thykamine™. All significant differences remained significant after Bonferroni adjustments. Error bars represent standard error of the mean. Six technical replicates per concentration per agent.

Concentration-dependent inhibition for both MIP-1α and MIP-1β was observed with all tested agents, except pimecrolimus (Figure 2A and Figure 3A). Thykamine™ showed higher inhibition than all other drugs at all concentrations for both MIP-1α and MIP-1β (Figure 2A and Figure 3A).

The mean AUC (SE) for percent inhibition of Thykamine™ for MIP-1α and MIP-1β was 2.45 (0.01) and 2.46 (0.01), respectively, and significantly higher versus all other drugs tested (*p* < 0.001) (Figure 2B and Figure 3B).

### 3.3. RANTES

**Figure 4:**
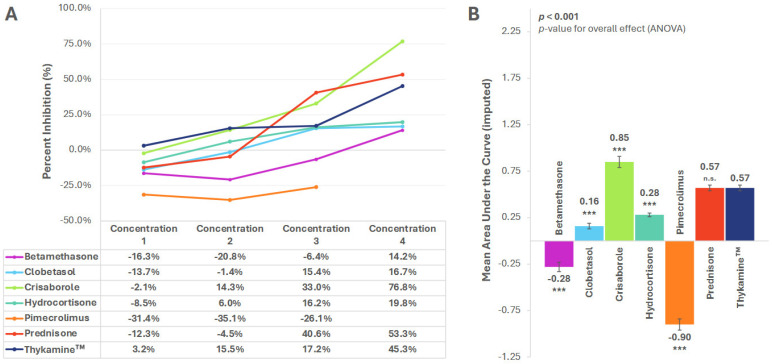
Inhibition of RANTES by anti-inflammatory agents. (**A**) Percent inhibition of RANTES secretion by anti-inflammatory agents across concentrations; (**B**) Mean area under the curve (AUC) for percent inhibition. AUC imputed for pimecrolimus. *** *p* < 0.001, n.s. = not significant. *p*-value for planned paired contrast (ANOVA) versus Thykamine™. All significant differences remained significant after Bonferroni adjustments. Error bars represent standard error of the mean. Six technical replicates per concentration per agent.

At the highest anti-inflammatory concentrations tested, secretion of RANTES was inhibited by all drugs tested, except pimecrolimus (Figure 4A). Thykamine™ inhibited RANTES secretion more potently than all other drugs, except at the two highest concentration levels of prednisone and crisaborole (Figure 4A).

Mean AUC analysis across concentrations showed that Thykamine™ [AUC (SE): 0.57 (0.03)] inhibited the release of RANTES significantly more than betamethasone [−0.28 (0.05), *p* < 0.001], clobetasol [0.16 (0.03), *p* < 0.001], hydrocortisone [0.28 (0.02), *p* < 0.001], pimecrolimus [−0.90 (0.06), *p* < 0.001], less than crisaborole [0.85 (0.06), *p* < 0.001], and similarly to prednisone [0.57 (0.03), *p* = 0.955] (Figure 4B).

## 4. Discussion

This study provides evidence that the clinical anti-inflammatory benefits from Thykamine™ observed in clinical trials likely result from Thykamine™ inhibiting the secretion of multiple key inflammatory chemokines. These chemokines collectively orchestrate the infiltration of monocytes, macrophages, neutrophils, T cells, and eosinophils into inflamed tissues. By dampening chemokine production, Thykamine™ limits both early innate and later adaptive inflammatory responses, thereby reducing leukocyte-driven cytokine release, oxidative stress, and tissue injury. Moreover, this study found that the in vitro molecular anti-inflammatory effect of Thykamine™ is comparable to or more robust than the six marketed anti-inflammatory agents tested.

Following LPS stimulation, Thykamine™ inhibition of three chemokines (MCP-1, MIP-1α, MIP-1β) was significantly greater compared to all six agents. In the case of RANTES, the inhibitory effect of Thykamine™ was greater than betamethasone, clobetasol, hydrocortisone, and pimecrolimus; not different from prednisone; and lower than crisaborole due to crisaborole’s elevated potency at higher concentration. At the lower concentrations, Thykamine™ consistently showed higher percent inhibition compared to all agents tested across all four chemokines assessed.

The increased chemokine secretion by LPS-stimulated monocytic cells following treatment with the calcineurin inhibitor pimecrolimus was within expectations. This relates to the fact that in monocytes and macrophages, calcineurin can serve as a brake on TLR signaling and reduce the activation of inflammatory transcription factors, such as nuclear factor kappa-light-chain-enhancer of activated B cells (NF-κB) and Y-box (YB) protein-1 [69,70]. Inhibition of calcineurin by pimecrolimus likely weakened this restraint, leading to enhanced activation of NF-κB and increased transcription of NF-κB-dependent chemokines, such as MCP-1, MIP-1α, MIP-1β, and RANTES. This observation provides validation of our study methodology.

The broad efficacy of Thykamine™ observed against the secretion of MCP-1, MIP-1α, MIP-1β, and RANTES suggests a multi-modal mechanism, attributable to the multi-component nature of the extract. This aligns with previous in vitro results [58] and the clinical efficacy observed for Thykamine™ in Phase II clinical trials for the treatment of mild-to-moderate atopic dermatitis [62] and UC [61]. By simultaneously modulating multiple chemokines rather than acting on a single target and offering antioxidant activity via contained pigments and enzymes [58], Thykamine™ may lower the risk of resistance and flare-ups that can occur with narrowly targeted agents, offering the potential for more durable disease control in chronic inflammatory settings. Based on the inhibitory effects on chemokines, Thykamine™ may act against inflammation by inhibiting the master regulators of inflammation, such as NF-κB, with downstream effects on numerous inflammatory pathways and targets. Given that the chemokines studied herein are commonly elevated across diverse autoimmune and inflammatory conditions, Thykamine™ may confer clinical benefit for a broad range of inflammatory indications beyond mild-to-moderate atopic dermatitis [62] and UC [61].

This study has limitations inherent to in vitro experiments, including possible differences between in vitro and in vivo inflammatory responses and the comparability of drug concentrations tested. Future studies should focus on expanding our understanding of the anti-inflammatory effects of Thykamine™, including on other inflammatory signaling molecules.

The favorable safety profile of Thykamine™ shown previously [62] combined with its potency observed in this study and others [58,61,62] provide strong support for investigating its potential as a novel and effective anti-inflammatory agent for conditions where chemokine-driven immune cell recruitment is central to disease pathogenesis. These include atopic dermatitis, rheumatoid arthritis, inflammatory bowel disease, and severe COVID-19.

In conclusion, Thykamine™ showed significantly greater or comparable inhibitory potency, particularly at lower concentrations, without evidence of cytotoxicity. These findings underscore the potential of Thykamine™ as a potent, multi-target anti-inflammatory therapy, which could offer substantial clinical advantages by effectively controlling chemokine-mediated inflammation with potentially fewer adverse effects. The results of this study support the need for evaluation of the clinical therapeutic efficacy of Thykamine™ in a wide range of autoimmune conditions.

## Figures and Tables

**Table 3:** Commonly prescribed anti-inflammatory agents.

Agent	Mechanism of Action	IC_50_	Route	Common Uses
Clobetasol propionate	Binds glucocorticoid receptor	CYP3A5: 206 nM [48] CYP3A4: 15.6 μM [48]	Topical, very high potency [49]	Corticosteroid-responsive dermatoses (e.g., atopic dermatitis, psoriasis, contact dermatitis)
Betamethasone valerate		MIP-1α: 38 nM [50]	Topical, high potency [49]	
21-acetate hydrocortisone		MIP-1α: 480 nM [50]	Topical, low potency [49]	
Prednisone		IL-5: 50 nM [51] IL-2: 200 nM [51]	Oral	Cancer, various inflammatory diseases (e.g., rheumatoid arthritis, atopic dermatitis, asthma, ulcerative colitis, Crohn’s disease, inflammatory bowel disease)
Crisaborole	Inhibits PDE4, causing increase in cAMP [52], which modulates NF-κB [53]	PDE4: 490 nM [54] TNF-α: 540 nM [54] IL-2: 610 nM [54] IFN-γ: 830 nM [54]	Topical	Mild-to-moderate atopic dermatitis
Pimecrolimus	Inhibits calcineurin, preventing NFAT dephosphorylation, preventing transcription of key cytokines and chemokines [55]	T-cell proliferation: 0.55 nM [56,57]	Topical	Mild-to-moderate atopic dermatitis

Numbers in square brackets correspond to published evidence. IC_50_: half-maximal inhibitory concentration; PDE4: Phosphodiesterase-4; TNF-α: Tumor Necrosis Factor-alpha; IL-2: Interleukin-2; IFN-γ: Interferon-gamma; MIP-1α: Macrophage Inflammatory Protein-1 alpha; IL-5: Interleukin-5; cAMP: Cyclic Adenosine Monophosphate; NF-κB: Nuclear Factor Kappa-Light-Chain-Enhancer of Activated B Cells; NFAT: Nuclear Factor of Activated T Cells.

**Table 4:** Formulations tested.

Agent Name	Concentration Levels				Manufacturer	Catalog
	1	2	3	4		
Thykamine™ (µg/mL)	125	250	500	1000	Devonian Health Group (Montmagny, QC, Canada)	N/A
Clobetasol propionate (nM)	0.1	1	10	50	Sigma-Aldrich	C8037
Betamethasone valerate (nM)	0.1	1	10	50	Sigma-Aldrich	PHR1780
21-acetate hydrocortisone (µM)	1	10	50	250	Sigma-Aldrich	H0888
Crisaborole (µM)	1	5	10	50	Cayman Chemical (Ann Arbor, MI, USA)	21455
Pimecrolimus (nM)	100	300	600	-	Sigma-Aldrich	SML1437
Prednisone (µM)	0.01	0.1	0.5	1	Sigma-Aldrich	P6254

RPMI: Roswell Park Memorial Institute; DMSO: Dimethyl Sulfoxide; N/A: Not Applicable.

## Data Availability

The data underlying this article will be shared upon reasonable request to the corresponding author.

## Abbreviations

ANOVA	Analysis of Variance
AUC	Area Under the Curve
CA	California
CCL	C-C Chemokine Ligand
CCR	C-C Chemokine Receptor
CO_2_	Carbon Dioxide
COVID-19	Coronavirus Disease 2019m
DMSO	Dimethyl Sulfoxide
LPS	Lipopolysaccharide
MCP-1	Monocyte Chemoattractant Protein-1
MIP-1α	Macrophage Inflammatory Protein-1 alpha
MIP-1β	Macrophage Inflammatory Protein-1 beta
NF-κB	Nuclear Factor Kappa-Light-Chain-Enhancer of Activated B Cells
ON	Ontario
PAMP	Pathogen-Associated Molecular Pattern
RANTES	Regulated on Activation, Normal T Cell Expressed and Secreted
RPMI	Roswell Park Memorial Institute
TLR	Toll-Like Receptor
UC	Ulcerative Colitis
USA	United States of America
VA	Virginia
YB	Y-Box
